# The reliability of pressure pain threshold in individuals with low back or neck pain: a systematic review

**DOI:** 10.1177/20494637231196647

**Published:** 2023-08-26

**Authors:** Anit Bhattacharyya, Lily D Hopkinson, Paul S Nolet, John Srbely

**Affiliations:** 1 Department of Population Medicine, Ontario Veterinary College, 3653 University of Guelph, Guelph, ON, Canada; 2 Faculty of Medicine, University of Ottawa, Ottawa, ON, Canada; 3 Department of Human Health & Nutritional Sciences, 3653 University of Guelph, Guelph, ON, Canada; 4 Department of Graduate Education and Research, 7948 Canadian Memorial Chiropractic College; 5 CAPHRI School for Public Health and Primary Care, Faculty of Health, Medicine, and Life Sciences, Maastricht University, Maastricht, The Netherlands

**Keywords:** Low back pain, neck pain, pain measurement, chronic pain, myofascialpain syndromes, musculoskeletal pain

## Abstract

**Background and Objective:**

Low-back and neck pain affect a great number of individuals worldwide. The pressure pain threshold has the potential to be a useful quantitative measure of mechanical pain in a clinical setting, if it proves to be reliable in this population. The objectives of this systematic review are to: (1) analyze the literature evaluating the reliability of pressure pain threshold (PPT) measurements in the assessment of neck and low-back pain, (2) summarize the evidence from these studies, and (3) characterize the limitations of PPT measurement.

**Databases and Data Treatment:**

Relevant literature from PubMed and the Web of Science electronic databases were screened in a 3-step process according to inclusion/exclusion criteria. Relevant studies were assessed for risk of bias using the Quality Appraisal of Reliability Studies (QAREL) tool, and results of all studies were summarized and tabulated.

**Results:**

Of 922 citations identified, 11 studies were deemed relevant for critical appraisal, and 8 studies were deemed to have low risk-of bias. Intra-rater reliability, reported in all studies (*n* = 637) and inter-rater reliability, reported in 2 studies (*n* = 200) were consistently reported to be good to excellent (ICC 0.75–0.99 and ICC 0.81–0.90, respectively). Studies were also found to have significant variation in PPT measurement procedures.

**Conclusions:**

Though intra- and inter-rater reliability was found to be high in all studies, the variation in PPT measurement protocols could affect validity and absolute reliability. As such, it is recommended that standard guidelines be developed for clinical use.

## Significance

This review provides a novel summation and quality appraisal of the current literature on the reliability of pressure pain threshold (PPT) as a quantitative pain assessment tool in a specific patient population. This work supports the development of guidelines to improve clinical use of PPT for patients with low back and neck pain by confirming its reliability and identifying its limitations.

## Introduction

Low-back and neck pain account for 75% of the total years lived with disability caused by musculoskeletal disease,^1^ and are challenging to treat even with combined therapies.^2,3^ Enhancing diagnosis and treatment of neck and back pain is crucial to improving quality of life for countless individuals, ultimately reducing the financial burden these conditions pose to society.^4^

Pain assessment tools enable clinicians to monitor the progression of these conditions. Since no valid and reliable biomarker for pain exists, most currently available tools rely on qualitative measures.^5^ Self-assessments, for example, are useful for their simplicity, but their subjective nature can introduce error.^6^ The need to shift pain assessment to a quantitative measure is crucial.

Pressure pain threshold (PPT), the point at which a pressure stimulus becomes painful,^7^ serves as a quantitative measure of pain.^8^ PPTs are measured using an algometer, which records the pressure applied to a given area as the subject notes the stimulus as painful. The desirable cost and short duration of time required to administer PPT are ideal; however, factors inherent to its measurement introduce the potential for bias error. PPT is a psychophysical measurement relying on perceptual input from the patient and proper technique by the observer.^8^ The variability in rate and angle of application between care providers also affects PPT values.^9,10^

Numerous studies have addressed PPT reliability in healthy and diseased individuals.^11–13^ Recent studies have provided further evidence of reliability in occupational groups such as vine-workers and office-workers, who are at high-risk of low-back and neck pain, respectively.^14,15^ However, there has yet to be any risk of bias assessments conducted to establish study quality.

This novel systematic review focuses on low-back and neck pain due to their disproportionate burden on the healthcare system as compared to other areas of non-specific pain.^16^ This focus increases the number of stakeholders, as these conditions are managed by many different healthcare professionals.^2,3^

The purpose of this study is to (1) analyze the literature evaluating reliability of PPT measurements in the assessment of low-back and neck pain, (2) to summarize the evidence from these studies, and (3) to identify limitations of PPT reliability. The results of this systematic review will help to better define the clinical treatment and management landscape for these non-specific and persistent syndromes and inform future studies investigating applications of PPT for clinical use.

## Methods

Studies were included with participants over 18 years of age presenting with pain affecting the low-back and neck. The anatomical back region is defined as the posterior aspect of the trunk inferior to the neck and superior to the gluteal region.^17^ The anatomical neck region connects the base of the skull to the torso and consists of the cervical portion of the spine.^18^ Since the aim of this study is to address relative reliability and not absolute reliability, confounding factors due to the type of pain are being considered negligible. The use of pain as a general term also allows us to explore PPT reliability as broadly as possible and highlight any niche areas in which PPT is less reliable.

### Eligibility criteria

Studies were included or excluded according to the following criteria. To be included in this systematic review, studies must have fulfilled the following criteria: (1) written in English; (2) published from January 1^st^, 2000 to January 1^st^, 2021; (3) published in a peer-reviewed journal; (4) manuscript available in full; (5) participants must be 18 years or older with a pain syndrome affecting the anatomical back or neck. For studies that also evaluated regions outside of the anatomical neck or back, results had to be stratified by anatomical location to be included; (6) PPT was measured using a standard algometer (manual or electronic) equipped with a rubber tip.

Studies that met any of the following criteria were excluded: (1) publication types including: books, commentaries, conference proceedings, consensus development statements, dissertations, editorials, government reports, guidelines, lectures and addresses, letters, and meeting abstracts; (2) studies containing less than 20 human participants; (3) studies with only healthy participants; (4) studies that did not contain appropriate measures of statistical agreement and did not publish data adequate enough to calculate these measures; (5) studies in which the measures of statistical agreement were only noted in the healthy control group and not those with pain symptoms.

### Data sources and searches

The search strategy was developed with the aid of a librarian that specializes in information literacy and research consultation. The electronic databases PubMed and Web of Science were searched for terms relevant to PPT of the back or neck and reliability, most recently on August 23, 2022 (Table 1). The Web of Science all database search function was used, thus incorporating nine additional databases, including but not limited to BIOSIS and SciELO (Table 2). Findings were extracted to Microsoft excel, and papers were included in the study if they met the inclusion–exclusion criteria.

**Table 1.** List of search parameters for PubMed database search and number of results.

Search field #	Search term	# Of Results	Search parameters
#1	PPT reliability Back	10	PPT [All Fields] AND reliability [All Fields] AND ("back"[MeSH Terms] OR "back"[All Fields])
#2	Pressure pain threshold reliability Back	17	("pressure"[MeSH Terms] OR "pressure"[All Fields]) AND ("pain threshold"[MeSH Terms] OR ("pain"[All Fields] AND "threshold"[All Fields]) OR "pain threshold"[All Fields]) AND reliability[All Fields] AND ("back"[MeSH Terms] OR "back"[All Fields])
#3	Algometer reliability Back	5	algometer [All Fields] AND reliability[All Fields] AND ("back"[MeSH Terms] OR "back"[All Fields])
#4	Algometry reliability Back	5	algometry [All Fields] AND reliability[All Fields] AND ("back"[MeSH Terms] OR "back"[All Fields])
#5	PPT reliability Spine	7	PPT[All Fields] AND reliability[All Fields] AND ("spine"[MeSH Terms] OR "spine"[All Fields])
#6	Pressure pain Threshold Reliability Spine	8	("pressure"[MeSH Terms] OR "pressure"[All Fields]) AND ("pain threshold"[MeSH Terms] OR ("pain"[All Fields] AND "threshold"[All Fields]) OR "pain threshold"[All Fields]) AND reliability[All Fields] AND ("spine"[MeSH Terms] OR "spine"[All Fields])
#7	Algometer reliability Spine	4	algometer[All fields] AND reliability[All fields] AND ("spine"[MeSH Terms] OR "spine"[All Fields])
#8	Algometry reliability Spine	1	algometry [All Fields] AND reliability [All Fields] AND ("spine"[MeSH Terms] OR "spine"[All Fields])
#9	PPT reliability Neck	7	PPT [All Fields] AND reliability [All Fields] AND ("neck"[MeSH Terms] OR "neck"[All Fields])
#10	Pressure pain threshold reliability Neck	15	("pressure"[MeSH Terms] OR "pressure"[All Fields]) AND ("pain threshold"[MeSH Terms] OR ("pain"[All Fields] AND "threshold"[All Fields]) OR "pain threshold"[All Fields]) AND reliability [All Fields] AND ("neck"[MeSH Terms] OR "neck"[All Fields])
#11	Algometer reliability Neck	7	algometer [All Fields] AND reliability [All Fields] AND ("neck"[MeSH Terms] OR "neck"[All Fields])
#12	Algometry reliability Neck	6	algometry [All Fields] AND reliability [All Fields] AND ("neck"[MeSH Terms] OR "neck"[All Fields])

### Study selection

**Figure 1.**
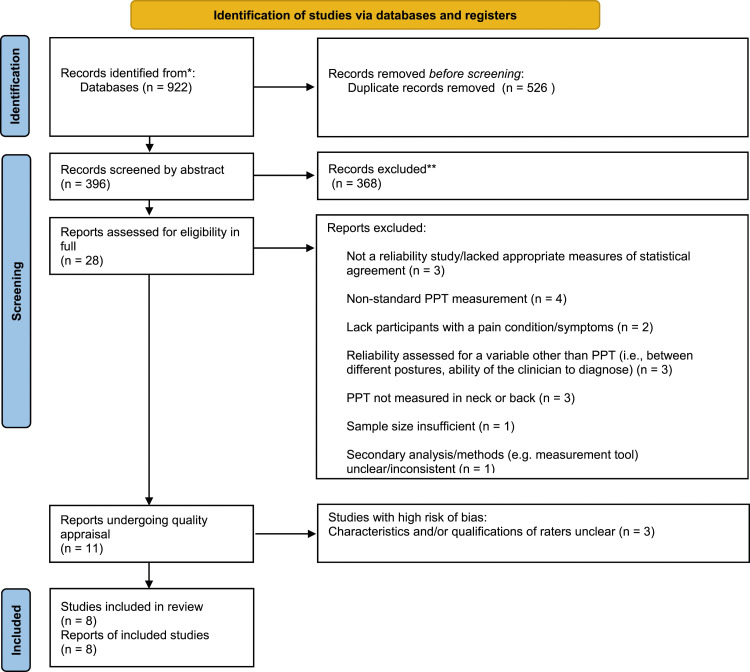
PRISMA 2020 flow diagram for new systematic reviews which included searches of databases and registers only.

Study selection was conducted in three stages (Figure 1). Stage 1 consisted of exporting search results to excel to identify duplicates. Duplicates were removed and articles were sorted into a table consisting of (1) title; (2) abstract; (3) rater evaluation. In stage 2, abstracts were screened by two raters (AB and LH) based on the inclusion/exclusion criteria, identifying papers as either relevant (Y), or irrelevant (N). Upon identification of relevant articles, we proceeded to stage 3, in which the raters revaluated the remaining articles in full. This evaluation followed the same relevant or irrelevant identification scheme. Comments were recorded for all irrelevant articles to track reasons for exclusion. At both stages, disagreements were discussed by evaluators (AB and LH) to reach consensus. Where consensus could not be met, a third independent evaluator was consulted (PN).

**Table 2.** List of search terms for web of science full archive database search and number of results.

Search field #	Search term	# Of results
#1	PPT AND Reliability AND Back	83
#2	“Pressure Pain Threshold” AND Reliability AND Back	192
#3	PPT AND Reliability AND Spine	58
#4	Pressure Pain Threshold AND Reliability AND Spine	106
#5	(Algometer OR Algometry) AND Reliability and (Spine OR Back)	106
#6	(Dolorimeter OR Dolorimetry) AND Reliability AND (Spine OR Back)	6
#7	PPT AND Reliability AND Neck	117
#8	“Pressure Pain Threshold” AND Reliability AND Neck	159
#9	(Dolorimeter OR Dolorimetry) AND Reliability AND (Neck)	3

### Assessment of risk of bias

Two reviewers (AB and LH) rated relevant studies (Table 3) using the Quality Appraisal of Reliability Studies (QAREL) checklist.^19^ The purpose of this tool is to standardize the evaluation of diagnostic reliability studies. Studies were then classified as low-risk or high-risk based on findings from the QAREL checklist, and high-risk studies excluded from the primary analysis. The relevance of each question in the QAREL evaluation was assessed by AB, JS, and PN prior to conducting the evaluation.

**Table 3.** Risk of bias for scientifically admissible reliability studies based on the QAREL criteria. Y – Yes, N – No, N/A – Not Applicable, UC – Unclear.

Author, year	Study objective clear defined	Representative sample	Representative raters	Blinded to Other’s findings	Blinded to own prior findings	Blinded to Ref standard	Blinded to clinical information	Blinded to other Clues	Order of Exam varied	Test applied correctly	Appropriate statistical measures
Balaguiler et al., 2016	Y	Y	Y	N/A	UC	N/A	UC	UC	UC	Y	Y
de Oliveira et al., 2021	Y	Y	Y	Y	UC	N/A	UN	UC	UC	Y	Y
Ferreia et al., 2020	Y	Y	Y	N/A	N	N/A	Y	UC	N	Y	Y
Jørgensen et al., 2014	Y	Y	Y	N/A	UC	N/A	N	Y	UC	Y	Y
Park et al., 2011	Y	Y	Y	N/A	UC	N/A	UC	UC	N	Y	Y
Prushansky et al	Y	Y	UC	UC	UC	N/A	UC	UC	UC	Y	Y
Sterling et al., 2002	Y	Y	UC	N/A	N	N/A	UC	UC	N	Y	Y
Walton et al., 2011	Y	Y	Y	Y	UC	N/A	Y	UC	Y	Y	Y
Wang-Price et al	Y	Y	UC	N/A	UC	N/A	UC	UC	UC	Y	Y
Ylinen et al., 2007	Y	Y	Y	N/A	Y	N/A	N	UC	N	Y	Y
Zicarelli et al., 2020	Y	Y	Y	N/A	UC	N/A	Y	UC	UC	Y	Y

### Summary of evidence

Both raters (AB and LH) collaborated to create a summary of evidence table, which reports the data items of interest extracted from each study (Table 3). The summary includes details about (1) study design (test-retest/intra-rater, inter-rater or both); (2) sample size; (3) case definitions and cohort details; (4) PPT measurement protocol; (5) examiner qualifications; (6) time between assessments; (7) relevant reliability statistics (intra-class correlation coefficient, Cronbach’s alpha); (8) study quality.

### Analyses

The primary analysis of this review excludes those studies which were identified to have high-risk of bias. A sensitivity analysis was performed that included the findings from studies found to have high risk-of-bias, to ensure the conclusions of the study are robust to this accommodation.

### Reporting compliance, protocol, and registration

This systematic review complies with the Preferred Reporting Items for Systematic Reviews and Meta-Analyses (PRISMA). A separate review protocol was not prepared but is well described in the preceding section of the manuscript. This review was not registered, though no systematic reviews of PPT reliability in our target population exist to our knowledge.

## Results

### Study selection

A total of 922 citations were identified through PubMed and Web of Science (full archive). Five hundred and twenty-six duplicates were removed leaving 396 citations for screening. In the primary screening stage, 368 citations were identified as ineligible, leaving 28 citations for full article screening. Seventeen (17) citations were deemed ineligible, leaving 11 citations for summary of evidence and critical appraisal. Findings from a total of *n* = 350 low-risk of bias subjects, and *n* = 119 high risk of bias subjects are summarized in Table 4.

**Table 4.** Evidence table for studies that assess the reliability of PPT measurements of the back and neck.

Authors, year Country	Design Sample size (n)	Case definition	Index test	Reliability
Low-risk studies				
Balaguiler et al., 2016^14^ France	Intra-rater reliability *n* = 29	LBP (*n* = 19 chronic, *n* = 3 acute) Healthy controls (*n* = 7) 25–60 years old	Hand-held electronic algometer with 1 cm2 tip was used for PPT measurement algometer was calibrated prior to data collection. 14 anatomical locations were tested 3 times each. Subjects were first familiarized by running a PPT test on tibialis anterior muscle. Subjects were given a button that locks the algometer reading and were told to press the button when the sensation turns from pressure to pain. Pressure was applied at 30 kPa/s perpendicular to the skin. Examiner: Masters in sport science with PPT training. Time between intra-rater assessments: 1 min between consecutive trials.	**Intra-rater reliability** ICC: 0.86–0.99 for all anatomical locations and all combinations
de Oliveira et al., 2021^27^ Brazil	Intra-rater and inter-rater reliability *n* = 100	Women with neck pain for more than 90 days (*n* = 30)	PPT measured via an algometer with a 1 cm^2^ rubber risk and constant speed of 0.5 kg/cm^2^/s. Pressure applied perpendicular to fibers of upper trapezius muscle bilaterally over trigger points. Examiner: Two examiners previously trained and familiarized with algometry. Time between assessments: One week apart	**Intra-rater reliability** Right upper trapezius ICC (95% CI): 0.752 (0.480–0.882) Left upper trapezius ICC (95% CI): 0.781 (0.609–0.877) **Inter-rater reliability** Right upper trapezius ICC (95% CI): 0.858 (0.778–0.914) Left upper trapezius ICC (95% CI): 0.874 (0.803–0.932)
Ferreira et al., 2020^25^ Portugal	Intra-rater *n* = 50	University students with idiopathic subclinical neck pain (*n* = 25) Healthy sex-matched participants (*n* = 25)	Standard algometer with 0.5 cm diameter tip, pressure applied at a rate of 3 N/s up to a maximum of 60 N Examiner: Two investigators trained by a third individual using a one-hour workshop, followed by a few days of training, and a pilot trial of 10 asymptomatic subjects (ICC 0.88 to 0.94 for all body parts) Time between assessments: 30 s	**Intra-rater reliability** Right C5-C6 ICC (95% CI): 0.79 (0.64–0.89) Left C5-C6 ICC (95% CI): 0.82 (0.71–0.92)
Jørgensen et al., 2014^23^ Denmark	Intra-rater reliability *n* = 42	Chronic neck pain (CNP) (*n* = 21) Healthy controls gender and age matched (*n* = 21)	Only examiner C performed PPT testing. Pressure was applied at a slow constant rate using an electronic hand-held algometer. Algometers were calibrated and subjects were given a training session prior to data collection Examiner: C – Experienced PT and manual therapist Time between assessments: Unclear (likely immediate).	**Intra-rater reliability** C3-C4 ICC (95% CI):0.89 (0.78–0.95) Infraspinatus ICC (95% CI): 0.83 (0.66–0.92)
Park et al., 2011^15^ Korea	Intra-rater reliability *n* = 222	Myofascial pain syndrome (*n* = 156) 43.2 ± 5.5 years old (mean)	PPT was measured using a digital algometer. Force was applied at a constant rate of 1 kg/cm^2^ across all 8 muscles then repeated 3 times. Measurements were made on predefined anatomical locations Examiner: Clinician with 10 years-experience Time between assessments: 5 min	**Intra-rater reliability** Cronbach’s a Upper trapezius Left: 0.980 Right: 0.939 Infraspinatus Left: 0.963 Right: 0.934
Walton et al., 2011^28^ Canada	Intra-rater and inter-rater reliability *n* = 100	Neck pain (*n* = 40) Healthy controls (*n* = 60)	Raters practiced algometry measurement on a firm surface until they were able to increase pressure at 50 kPa/s or 5 N/s Rater’s gave verbal instructions to participants, then used a handheld digital algometer with a 1 cm^2^ round rubber tip to measure PPT on the upper fibers of the trapezius muscle in seated position and on the muscle belly of tibialis anterior while lying down with knees flexed 90°. Measurements were taken 3 times at each site on each side of the body. Examiners: Novice raters (male physiotherapy students or clinical physiotherapists) trained to perform algometry. Time between assessments Intra-rater: 1 min Inter-rater: 3–5 min	**Intra-rater reliability – Control** Upper trapezius ICC (95% CI): 0.97 (0.94–0.98) Intra-rater reliability – Symptomatic Upper trapezius ICC (95% CI): 0.96 (0.91–0.98) **Inter-rater reliability – Control** Upper trapezius ICC (95% CI): 0.79 (0.66–0.87) **Inter-rater reliability - symptomatic** Upper trapezius ICC (95% CI): 0.81 (0.67–0.90)
Ylinen et al., 2007^26^ Finland	Intra-rater reliability *n* = 20	Middle-agedwomen with non-specific neck pain of at least 6 months duration	Hand-held digital algometer using a 1 cm2 tip at a rate of 10 N/s. Pressure was applied perpendicularly to the muscle Examiner: Physiotherapist with several years experience Time between assessments: 30 s and again 1 day later	**Intra-rater reliability** Right splenius capitis ICC (95% CI): 0.93 (0.83–0.97) Left splenius capitis ICC (95% CI): 0.84 (0.64–0.93) Right trapezius capitis ICC (95% CI): 0.86 (0.70–0.94) Left trapezius capitis ICC (95% CI): 0.85 (0.67–0.94) Right levator scapulae capitis ICC (95% CI): 0.78 (0.53–0.91) Left levator scapulae capitis ICC (95% CI): 0.91 (0.79–0.96)
Zicarelli et al., 2020^29^ Brazil	Intra-rater reliability *n* = 74	LBP (*n* = 21) Healthy controls gender and age matched (*n* = 21) Neck pain (*n* = 15) Healthy controls gender and age matched (*n* = 17) All 18–40 years old	PPT was measured using a calibrated electronic algometer with a 1 cm^2^ diameter tip administered perpendicular to the skin surface. PPT was measured bilaterally on sub-occipital, trapezius and supraspinatus muscles, as well as on the erector spinae muscle group. The rater was blinded to the participant group Examiner: Examiner with more than 5 years of clinical experience Time between assessments: 24 h	Neck pain ICC (95% CI): 0.87 (0.62–0.96) Neck control ICC (95% CI): 0.89 (0.71–0.96) Low back pain ICC (95% CI): 0.93 (0.83–0.97) Low back control ICC (95% CI): 0.84 (0.60–0.93)
High-risk studies				
Sterling et al., 2002^20^ Australia	Unclear *N* = 38	Chronic neck pain (*n* = 19) Asymptomatic (*n* = 19)	Electronic digital algometer was used with a 1 cm^2^ round tip. PPT was applied at a rate of 40 kPa/s. PPTs were measured bilaterally to the cervical spine at C2/3 articular pillars, and C5/6 vertebral segments Time between assessment: 1 week	Left C2/3 Neck pain ICC: 0.72 Asymptomatic ICC: 0.80 Right C2/3 Neck pain ICC: 0.89 Asymptomatic ICC: 0.80 Left C5/6 Neck pain ICC: 0.88 Asymptomatic ICC: 0.91 Right C6/6 Neck pain ICC: 0.92 Asymptomatic ICC: 0.88
Wang-Price et al., 2019^21^ United States	Unclear *n* = 60	Neck-shoulder pain and tenderness (*n* = 30) Asymptomatic (*n* = 30) *N* = 18 neck-shoulder pain and 20 asymptomatic individuals returned for the between-day reliability testing	Handheld computerized algometer with a 1 cm^2^ round tip was used. Pressure was applied vertically at a rate of 40 kPa/s. A limit of 600 kPa was used. The order of testing position is randomized, however, the muscle groups are always tested in the same order. Fourmeasurements were taken at each site, with at least 5 s between measurements, and the first being a practice trial Time between assessments: Minimum of 5 s for same-day and 3 to 7 days for between-day ratings	Within-day middle deltoid Neck pain ICC (95% CI) (seated/prone): 0.96 (0.93–0.98)/0.95 (0.92–0.98) Asymptomatic ICC (95% CI) (seated/prone): 0.93 (0.88–0.96)/0.91 (0.85–0.95) Within-day levator scapulae Neck pain ICC (95% CI) (seated/prone): 0.95 (0.92–0.98)/0.92 (0.86–0.96) Asymptomatic ICC (95% CI) (seated/prone): 0.95 (0.90–0.97)/0.94 (0.90–0.97) Within-day upper trapezius Neck pain ICC (95% CI) (seated/prone): 0.93 (0.88–0.96)/0.95 (0.91–0.98) Asymptomatic ICC (95% CI) (seated/prone): 0.85 (0.75–0.92)/0.90 (0.82–0.95) Between-day middle deltoid Neck pain ICC (95% CI) (seated/prone): 0.97 (0.91–0.99)/0.98 (0.95–0.99) Asymptomatic ICC (95% CI) (seated/prone): 0.73 (0.31–0.89)/0.86 (0.65–0.96) Between-day levator scapulae Neck pain ICC (95% CI) (seated/prone): 0.95 (0.86–0.98)/0.93 (0.82–0.98) Asymptomatic ICC (95% CI) (seated/prone): 0.91 (0.78–0.96)/0.89 (0.73–0.96) Between-day upper trapezius Neck pain ICC (95% CI) (seated/prone): 0.86 (0.64–0.95)/0.94 (0.82–0.98) Asymptomatic ICC (95% CI) (seated/prone): 0.71 (0.28–0.88)/0.87 (0.68–0.95)
Prushansky et al., 2007^22^ Israel	Test-retest and inter-rater. (*n* = 21)	Chronic whiplash (*n* = 21)	A handheld pressure algometer was used. Details regarding the methods were provided in a previous paper, however, a citation was not specified Time between assessments: 15 min for inter-rater; and test-retest 7.9 days apart	Test-retest C2 ICC right/left: 0.85/0.86 C4 ICC right/left: 0.90/0.91 C6 ICC right/left: 0.90/0.89 Total ICC: 0.91 Inter-rater C2 ICC right/left: 0.88/0.93 C4 ICC right/left: 0.90/0.96 C6 ICC right/left: 0.97/0.96

The results of the study selection process are thoroughly summarized in Figure 1.

### Critical appraisal

Eleven (11) studies were critically appraised for risk of bias using the QAREL evaluation tool. Three studies^20–22^ were deemed to have high-risk of bias because the characteristics and/or qualifications of the raters were unclear. Information regarding representative raters was also missing from the article by Balaguier et al.,^14^ however, when contacted for more information, authors clarified rater’s qualifications, and raters were deemed to be representative. The remaining studies were considered to have low risk of bias.

### Study characteristics

Six studies evaluated only test-retest reliability.^14,15,23–26^ Two studies evaluated both test-retest and inter-rater reliability.^27,28^ One study was conducted in each of France, Portugal, Denmark, Korea, Canada, and Finland. Two studies were conducted in Brazil. All studies were published between 2007 and 2020, and no studies were excluded due to their year of publication. One study recorded PPT from patients with low-back pain,^14^ five studies assessed patients with neck pain,^23,25–28^ one study assessed patients with either low-back or neck pain,^24^ and one study assessed patients with myofascial pain syndrome in the cervical spine and back regions.^15^

All studies recorded PPT measures using a digital algometer; however, procedures were not consistent. The size of the rubber tip varied, with five studies using a 1 cm^2^ tip (*n* = 5),^14,26–29^ one using a 0.5 cm^2^ tip (*n* = 1),^25^ while two groups did not state the size of the tip (*n* = 2).^15,23^ As variability in tip size remains consistent within each study, bias is prevented, and tip size did not disqualify a study. Additionally, all studies, regardless of tip size, were found to have good to excellent reliability, further supporting the conclusion that tip size did not have an impact on reliability.

Algometry was performed at a constant rate within each study (*n* = 8),^14,15,23–28^ but the rate at which it was applied between studies varied. Two studies administered pressure at a rate of 30 kPa/s (*n* = 2) (Ferreira et al. originally reported as 3 N/s),^14,25^ another at 49 kPa/s (reported as 0.5 kg/cm^2^/s),^27^ another at 50 kPa/s (*n* = 1),^28^ while two studies administered pressure at a rate of 1 kg/cm^2^/s (98 kPa/s) (*n* = 2),^15,24^ and the last at 100 kPa/s (originally reported as 10 N/s).^26^ One study did not report the rate of pressure application (*n* = 1)^23^ but we did not exclude it as relative reliability would not be affected since the rate was consistent within the study. All studies used qualified raters of varying levels of experience, with seven studies using healthcare providers as raters (*n* = 7),^14,15,23–27^ and one using a combination of a clinician and physiotherapy students, all of which were novice raters (*n* = 1).^28^ Every rater was representative of those who typically utilize PPT in a clinical setting.

The time between measurements also varied. In studies evaluating test-retest reliability, time between measurements ranged from 30 s,^25,26^ to 1 week.^27^ One study did not clearly report the time between measurements, but it was inferred based on the description of the test procedure that measures were repeated immediately (*n* = 1).^23^ For inter-rater reliability, the time between measurements was 3–5 min for one study,^28^ and one week for another.^27^ The differences in rest period were not considered to be of concern if the time between measurements was consistent for every patient within a study.

### Assessment of risk of bias

Studies that were considered to have low risk of bias adhered to the following criteria: (1) study objective clearly defined; (2) representative sample; (3) representative raters; (4) blinded to the findings of other raters; (5) test applied correctly; (6) appropriate statistical measurements. There were some limitations to the high-quality studies, as follows: (1) lacked blinding to own prior findings (*n* = 7)^14,15,23–25,27,28^; (2) no blinding to clinical information (*n* = 5);^14,15,23,26,27^ (3) no blinding to additional cues (*n* = 7);^14,15,24–28^ (4) non-random test administration (*n* = 8).^14,15,23–27^ Studies that did not clearly answer a particular question (NC) were counted as “no” for the purposes of limitation quantification. A summary of the risk of bias assessment can be found in Table 3.

### Test-retest reliability

Eight studies evaluating test-retest reliability were found to have high degrees of reliability. ICCs ranged from 0.75^27^ to 0.99^14^ demonstrating good to excellent reliability across the back and neck.^30^ Minimal differences in ICCs were observed between individuals with low-back (0.86–0.99)^14,24^ and neck pain (0.75–0.96)^27,28^ suggesting that PPT reliability did not change based on the location of pain or measurement. While studies that used patients with back pain had a slightly larger range of variability, at the extremes their ICCs were still rated as good or excellent.^14^ One study utilized Cronbach’s α as a statistical measure of reliability, with values ranging from 0.934 to 0.980.^15^ This range indicates a high degree of reliability^31^ and is consistent with the observations from other studies.

### Inter-rater reliability

Inter-rater reliability evaluations were only conducted in two studies addressing neck pain (*n* = 2).^27,28^ ICCs in symptomatic individuals were reported to be between 0.81^28^ and 0.86.^27^ This indicates good to excellent reliability in the measurement of neck pain.^30^

### High-risk of bias findings

Of the high risk of bias studies, ICCs corresponding to test-retest reliability ranged from 0.71^21^ to 0.98^21^ indicating a moderate to high degree of reliability.^30^ For two of the studies,^20,21^ it was unclear whether they used the same rater for both measurements. ICCs corresponding to inter-rater reliability, clearly reported in one study,^22^ were also excellent. All three studies reported good to excellent reliability in all but four measurements,^20–22^ and their findings are summarized in Table 4. These findings support the results of the low risk of bias studies.

## Discussion

Patients with low-back or neck pain contribute significantly to the total YLD.^16^ The burden on these patients is high and clinical outcomes remain poor.^2,3^ Improvements in pain assessment tools enable healthcare professionals to diagnose and treat these individuals more effectively, as their progress can be more accurately monitored. The PPT is a cost effective, clinically feasible assessment outcome with features that make it easy to adopt within a clinical setting for the management of musculoskeletal pain. This is the first systematic review to our knowledge to assess the reliability of PPT in neck and back pain subjects. The findings of this review suggest that PPT is a useful and reliable tool in clinical practice for pain assessment to monitor patient progress. This measurement can also contribute to standardization of pain assessment across providers using a psycho-physical outcome over the commonly used subjective pain scale.^8^ These findings also support the continued use of PPT in quantitative sensory testing,^32^ a reliable tool used in the measurement of two clinically correlated syndromes: myofascial pain syndrome,^33,34^ and central sensitization.^35,36^ This puts PPT in an adventitious position to play a larger role in the treatment and management of low-back and neck pain.

### Strengths and limitations

Our systematic review had several strengths. First, the PubMed search was conducted with the aid of a specialist in information literacy to ensure our search was as broad as possible. This ensured papers that had some relevant data could still be included in the review if the results were stratified properly. Additionally, the study quality assessment was conducted using a validated guideline (QAREL) and the risk of bias assessment was guided by multiple experts in the field. Lastly, the screening steps, as well as the QAREL assessment, were conducted by two raters to minimize potential bias.

The findings of this review should be interpreted in the context of several limitations. Only two search engines were used, which leaves the possibility that unindexed low-risk papers may have been missed. Given the low degree of variability across papers which met the quality criteria, it is unlikely this has a large impact on the findings. Additionally, risk from this has been mitigated using the Web of Science extended database search which included nine additional databases. The search strategy did not specify specific neck or back syndromes, in order to identify all relevant studies. Secondly, only papers in the English language were considered. PPT reliability studies in other languages may exist and would have been excluded. Key PPT papers that were found were published in English, and the low degree of variability in the findings of listed studies suggest it is unlikely studies in another language would largely affect the findings of this study. Two articles that were found in our initial search but were excluded due to the language requirement would have been excluded as irrelevant to the research question upon further investigation. Thirdly, our search excluded studies published prior to January 1^st^, 2000. This was done for practicality reasons, however, numerous studies from before 2000 also found a high degree of reliability for PPT,^37–39^ thus any relevant articles from before 2000 are unlikely to skew our findings. Additionally, standardization of algometers technique, and the availability of standardized algometers (of which many are still available for sale) has expanded drastically, thus studies from 2000 onwards offers an added level of consistency in instrumentation. Although using the pre-determined methodology and QAREL analysis indicated a high degree of reliability, the continued exploration of inter-rater reliability given the initial findings from this study is a recommendation of the authors. Another assumption of our study was that the pain response to rate of pressure applied is linear. The rate response curve has not been established to the best of our knowledge, however, only studies that used a constant rate of pressure application were included. Many of the studies included also have small sample sizes which can introduce bias. Given the consistency of findings between studies with highly variable sample sizes, it is unlikely to have a major impact on our study, although a meta-analysis and larger scale studies are recommended in future investigations. Finally, the QAREL assessment guidelines were determined by expert opinion in addition to consulting current literature, however, there is little to no literature regarding the limitations of the risk of bias assessment that was described above.

## Conclusion

The findings of this study demonstrate that PPT has a high degree of intra- and inter-rater reliability in individuals experiencing low-back or neck pain in a variety of clinical settings. Further studies describing the reliability and validity of PPT in different pain syndromes will help to further identify the optimal role of PPT in clinical settings. Additionally, given the variability across studies included in this systematic review, it is recommended that standard guidelines be developed to address (1) size and material of tip; (2) rate of pressure application; (3) angle of application, and (4) anatomical identification of measurement points. Although our systematic review has demonstrated that intra- and inter-rater reliability remains high regardless of these variations, these may affect absolute reliability and validity. We also recommend further exploration of inter-rater reliability of PPT given the high degree of reliability initially demonstrated. Future studies should investigate the validity of PPT. Standardization of PPT application will contribute to the timely and urgent priority of developing accurate and reliable biomarkers and reference standards in pain management.
